# Blockade of Receptor-Activated G_i_ Signaling in Osteoblasts In Vivo Leads to Site-Specific Increases in Cortical and Cancellous Bone Formation

**DOI:** 10.1002/jbmr.273

**Published:** 2010-10-11

**Authors:** Susan M Millard, Alyssa M Louie, Lalita Wattanachanya, Thomas J Wronski, Bruce R Conklin, Robert A Nissenson

**Affiliations:** 1 Endocrine Research Unit, Veterans Administration Medical Center and Departments of Medicine and Physiology, University of California San FranciscoSan Francisco, CA, USA; 2 Endocrinology and Metabolism Unit, Department of Medicine, King Chulalongkorn Memorial Hospital, Thai Red Cross Society and Faculty of Medicine, Chulalongkorn UniversityBangkok, Thailand; 3 Department of Physiological Sciences, University of FloridaGainesville, FL, USA; 4 Gladstone Institute of Cardiovascular DiseaseSan Francisco, CA, USA; 5 Department of Medicine, University of California San FranciscoSan Francisco, CA, USA; 6 Department of Cellular and Molecular Pharmacology, University of California San FranciscoSan Francisco, CA, USA

**Keywords:** OSTEOBLASTS, G PROTEIN–COUPLED RECEPTORS, G_i_ SIGNALING, PERIOSTEAL BONE FORMATION, SEXUAL DIMORPHISM, INBRED MICE

## Abstract

Osteoblasts play a critical role in the maintenance of bone mass through bone formation and regulation of bone resorption. Targeted expression of a constitutively active engineered G_i_-coupled G protein–coupled receptor (GPCR) to osteoblasts in vivo leads to severe osteopenia. However, little is known about the role of endogenous receptor-mediated G_i_ signaling in regulating osteoblast function. In this study, we investigated the skeletal effects of blocking G_i_-coupled signaling in osteoblasts in vivo. This was accomplished by transgenic expression of the catalytic subunit of pertussis toxin (PTX) under control of the collagen Iα 2.3-kb promoter. These mice, designated *Col1(2.3)*^*+*^*/PTX*^*+*^, showed increased cortical thickness at the femoral midshaft at 12 weeks of age. This correlated with increased periosteal bone formation associated with expanded mineralizing surface observed in 8-week-old mice of both genders. The cancellous bone phenotype of the *Col1(2.3)*^*+*^*/PTX*^*+*^ mice was sexually dimorphic, with increases in fractional bone volume at the distal femur seen only in females. Similarly, while cancellous bone-formation rates were unchanged in males, they could not be quantified for female *Col1(2.3)*^*+*^*/PTX*^*+*^ mice owing to the disorganized nature of the labeling pattern, which was consistent with rapid formation of woven bone. Alterations in osteoclast activity did not appear to participate in the phenotype. These data demonstrate that G_i_-coupled signaling by GPCRs endogenous to osteoblasts plays a complex role in the regulation of bone formation in a manner that is dependent on both gender and the anatomic site within bone. © 2011 American Society for Bone and Mineral Research.

## Introduction

G protein–coupled receptors (GPCRs) are the largest family of cell surface receptors, and as such, they mediate a host of cellular responses. GPCRs have been very fruitful therapeutic targets, with over 25% of drugs approved by the Food and Drug Administration acting directly on members of this receptor family.(1) GPCRs continue to be common targets of newly developed pharmaceuticals, and furthering our understanding of the biologic role of GPCRs still holds promise for the development of new therapeutics. Four families of Gα proteins are critical in mediating signaling downstream of GPCR activation: G_s_, G_i_, G_q_, and G_12_.(2) A role for G_s_-coupled signaling in osteoblasts in promoting trabecular bone formation and cortical bone resorption has been described in vivo in both gain-of-function and loss-of-function models.(3–6) The role of the other families of Gα proteins in the regulation of skeletal homeostasis remains less clear.

The classic downstream effector of G_i_ activation is the inhibition of adenylyl cyclase,(2) which would be expected to antagonize G_s_-mediated increases in adenylyl cyclase activity in osteoblasts. Transgenic expression of a constitutively active G_i_-coupled GPCR in osteoblasts leads to marked trabecular osteopenia,(7) consistent with the concept of G_i_-coupled signaling opposing G_s_-mediated trabecular bone formation. However, the role of endogenous G_i_-coupled receptors in regulating osteoblast function is less clear. A range of ligands has been shown to elicit G_i_-dependent mitogenic responses from osteoblastic cells in vitro. These ligands include fluoride,(8) lysophosphatidic acid,(9) and spingosine 1-phosphate.(10) Additionally, activation of the G_i_-coupled apelin receptor stimulates proliferation and inhibits apoptosis of human osteoblasts in vitro.(11,12) Such mitogenic effects may be mediated by alternative effectors of G_i_-coupled signaling such as mitogen-activated protein kinases (MAPKs) and phosphoinoisitide-3 kinase (PI3K).(13,14) Two candidate GPCRs have been suggested as potential targets for osteoporosis therapeutic strontium ranelate(15,16): the calcium-sensing receptor (CaSR)(17,18) and the closely related GPRC6A.(19) Both these GPCRs can couple with both G_i_ and G_q_,(20–22) suggesting that the in vivo anabolic actions of strontium could, at least in part, be mediated by stimulation of G_i_ signaling.

Genetic manipulation of endogenous G_i_-coupled GPCRs in mice has given rise to a range of skeletal phenotypes. Recently, the G_i_-coupled serotonin receptor Htr1b has been described as a negative regulator of osteoblast proliferation, with global and osteoblast-specific gene deletion of *Htr1b* leading to increased vertebral bone volume associated with increased osteoblast number.(23) The global knockout of the NPY Y1 receptor also shows increased cancellous bone volume that is proposed to result from loss of osteoblastic Y1 expression.(24) Conversely, deletion of the gene encoding the G_i_-coupled CB2 cannabinoid receptor led to accelerated age-related bone loss, and a CB2-specific agonist was found to attenuate bone loss induced by estrogen deficiency in mice.(25) There are, of course, limitations to interpreting phenotypes resulting from global gene deletions, and the marked difference in the skeletal phenotypes of the *Htr1b*^−/−^ and *CB2*^−/−^ mice may be due in part to differences in expression of these two receptors both within the osteoblast lineage and in other cell types. Additionally, the contexts in which osteoblasts are exposed to the ligands for these two receptors may differ significantly. Furthermore, if osteoblasts at different stages of the osteoblast lineage have altered responses to G_i_-coupled signaling, this potentially could reconcile the mitogenic effects of activation of G_i_-coupled receptors on osteoblastic cells in vitro with the restriction of osteoblast proliferation by the G_i_-coupled Htr1b in vivo.

In this study, we have pursued a strategy of osteoblast-specific blockade of G_i_-coupled signaling in order to further elucidate the role of this signaling pathway in regulating osteoblast function. G_i_-coupled signaling is mediated by a family of Gα_i_ subunits (Gα_i1_, Gα_i2_, Gα_i3_, Gα_o_, Gα_z_, the retinal α subunit Gα_t_, and the taste α subunit Gα_gust_)(2) and so cannot easily be eliminated using gene-deletion technology. However, GPCR-mediated activation of the most ubiquitous and abundant Gα_i_ subunits (Gα_i1_, Gα_i2_, Gα_i3_, and Gα_o_) is inhibited by pertussis toxin, and this agent therefore is used widely in vitro as a tool to identify G_i_-mediated effector responses. Here we have used osteoblast-specific expression of the catalytic subunit of pertussis toxin in vivo to probe the role of receptor-mediated G_i_ signaling in these cells.

## Materials and Methods

### Mouse studies

All transgenic mouse studies were approved by and performed in accordance with the Institutional Animal Care and Use Committee at the Veterans Affairs Medical Center, San Francisco, and at the University of California San Francisco. *Col1(2.3)-tTA* mice (line 139) have been described previously, and heterozygous *Col1(2.3)-tTA* mice have been shown to be indistinguishable from wild-type mice.(7) We have generated the transgenic mouse line that expresses the catalytic subunit of pertussis toxin (PTX) under regulatory control of the tetracycline transactivator (tTA)–responsive *tetO* promoter. The coding sequence for PTX (*Bordetella pertussis* strain HAV; Genbank No. AJ007364.1) was cloned into the tTA-inducible pUHG 10-3 (*tetO-PTX*). A purified *tetO-PTX* DNA fragment was prepared and used to microinject FVB/N oocytes. Injections were carried out at the Transgenic Core Facility of the UCSF-affiliated Gladstone Foundation, according to their standard methods. Polymerase chain reaction (PCR) from tail genomic DNA was used to screen for founders (using the primers CCA TAG AAG ACA CCG GGA CCG and GGA ACG TCC GGT CAG ATG GTC GA). A single founder was identified and backcrossed with wild-type FVB/N mice for more than six generations. *TetO-PTX* mice were transferred subsequently to the Transgenic Mouse Facility at the Veterans Affairs Medical Center, San Francisco. The *Col1(2.3)*^*+*^*/PTX*^*+*^ mice and *Col1(2.3)-tTA* control littermates used in this study were generated by crossing mice heterozygous for the *tetO-PTX* transgene with mice that were true breeding for the *Col1(2.3)-tTA* transgene. All animals were maintained on the FVB/N background. Mice were maintained on either standard mouse chow or, where indicated, a diet containing 200 mg/kg of doxycycline (DoxDiet, Bio-Serv, Frenchtown, NJ, USA).

### RNA extraction and RT-qPCR

Tissue samples were isolated and kept frozen in liquid nitrogen until processing. Prior to freezing, epiphyses were removed and bone marrow flushed from femoral bone samples. Frozen tissues were pulverized using a biopulverizer (Biospec Products, Inc., Bartlesville, OK, USA), followed by RNA extraction using RNA-STAT60 (Tel-Test, Inc., Friendswood, TX, USA) and subsequent purification using Micro-to-Midi Total RNA Purification Kit (Invitrogen, Carlsbad, CA, USA). cDNA was synthesized using TaqMan Reverse Transcription Reagents (Applied Biosystems, Inc., Foster City, CA, USA) and random hexamer primers according to the recommendations of the manufacturer. Gene amplification was measured with either SYBR Green or Taqman chemistry using the ABI Prism 7300 real-time thermocycler (Applied Biosystems, Inc.). Analysis was carried out using the SDS software supplied with the thermocycler using both *Gapdh* and *L19* as the calibrator gene. In all cases, the choice of calibrator gene did not influence the data. All data are displayed normalized to *Gapdh*. Primer details are listed in the supplemental material.

### Micro–computed tomography (µCT)

Left femurs from 3-, 8-, and 12-week-old mice were isolated and cleaned of adherent tissue. Before µCT analysis, bones were fixed for 1 to 2 days in 10% neutral buffered formalin (NBF; Fisher Scientific, Pittsburgh, PA, USA) and dehydrated in 70% ethanol. The femurs were imaged using a vivaCT-40 µCT system (Scanco Medical AG, Bruttisellen, Switzerland). Imaging of cancellous bone was carried out at the distal metaphysis, and imaging of diaphyseal cortical bone was carried out at the midpoint of the femur. All images were obtained using an X-ray energy of 55 kV with a voxel size of 10.5 µm and integration time of 1000 ms. Two regions of interest (ROIs) were chosen for assessment of cancellous bone. ROI1 specifies a volume of metaphyseal cancellous bone from immediately adjacent to the primary spongiosa to a distance of 1.05 mm from the primary spongiosa. ROI2 specifies a volume of metaphyseal cancellous bone at a distance of 1.05 to 2.10 mm from the primary spongiosa. These cancellous ROIs were assessed in femurs from 8- and 12-week-old mice using a global thresholding protocol with segmentation values of 0.4/1/270. Quantitative assessment of diaphyseal cortex was conducted using data from 40 slices (0.42 mm) at the midfemur. Cortical bone from 3-week-old mice was assessed using a global thresholding protocol with segmentation values of 0.8/1/300. Segmentation values of 0.8/1/365 were used in the assessment of cortical bone from 8- and 12-week-old mice.

### Quantitative static histomorphometry

Femurs from 12-week-old mice were isolated at the time of euthanasia and fixed in 10% NBF for 1 to 2 days and stored in 70% ethanol. Following µCT analysis, the undecalcified bone samples were embedded in methyl methacrylate and sectioned with Jung 2065 and 2165 microtomes (Leica, Bannockburn, IL, USA). Osteoblast and osteoclast surfaces in the secondary spongiosa of the distal femoral metaphysis were measured in von Kossa/tetrachrome-stained sections with an Osteomeasure System (Osteometrics, Decatur, GA, USA) as described previously.(26)

### Dynamic histomorphometry

Mice were injected with 20 mg/kg of calcein (Sigma-Aldrich, St Louis, MO, USA) 21 and 7 days before euthanasia and with 15 mg/kg of demeclocycline (Sigma-Aldrich) 2 days before euthanasia. Mice were euthanized at 8 weeks, and femurs were isolated, fixed in 10% NBF, and stored in 70% ethanol. Following µCT analysis, the undecalcified bone samples were embedded in methyl methacrylate. Assessment of cancellous bone was performed on 10-µm longitudinal sections from the left femur. Assessment of cortical bone was performed on 10-µm transverse sections at the midpoint of the right femur. Mosaic-tiled images were acquired at × 20 magnification with a Zeiss Axioplan Imager M1 microscope (Carl Zeiss MicroImaging, Thornwood, NY, USA) fitted with a motorized stage. The tiled images were stitched and converted to a single image using the Axiovision software (Carl Zeiss MicroImaging) prior to blinded analyses being performed using image-analysis software (Bioquant Image Analysis Corp., Nashville, TN, USA). Cancellous bone was assessed in an irregular ROI defined by four boundaries: a line traced at a distance of 500 µm from the growth plate, two lines drawn parallel to the cortical bone at a distance of 200 µm from the endocortical surface, and a straight line drawn perpendicular to the bone at a distance of 1.7 mm from the lowest point of the growth plate. An average of 5.3 mm^2^ of cancellous bone tissue (including marrow) was measured. The dynamic indices of bone formation that were measured include mineralizing surface, mineral apposition rate, and surface-based bone-formation rate (BFR/BS).

### Serum chemistry

Blood was collected from mice at the time of euthanasia and processed in MicroTainer serum separator tubes (BD Biosciences, San Jose, CA, USA). Serum procollagen type I amino-terminal propeptide (PINP) and serum pyridinoline measurements were carried out using the rat/mouse PINP EIA Kit AC-33F1 from Immunodiagnostic Systems (Scottsdale, AZ, USA) and the MetraBiosystems SerumPYD Kit 8019 (MetaBiosystems, Santa Clara, CA, USA) according to manufacturers' directions.

### Statistical analysis

All data are presented as means ± SD. Statistical significance was ascertained by comparison between *Col1(2.3)-PTX* mice and sex-matched littermate controls using a two-tailed Student's *t* tests assuming equal variance or, where indicated, by two-way ANOVA.

## Results

### Generation of mice with osteoblast-specific, tetracycline-regulated expression of the catalytic subunit of pertussis toxin PTX

**Fig. 1:**
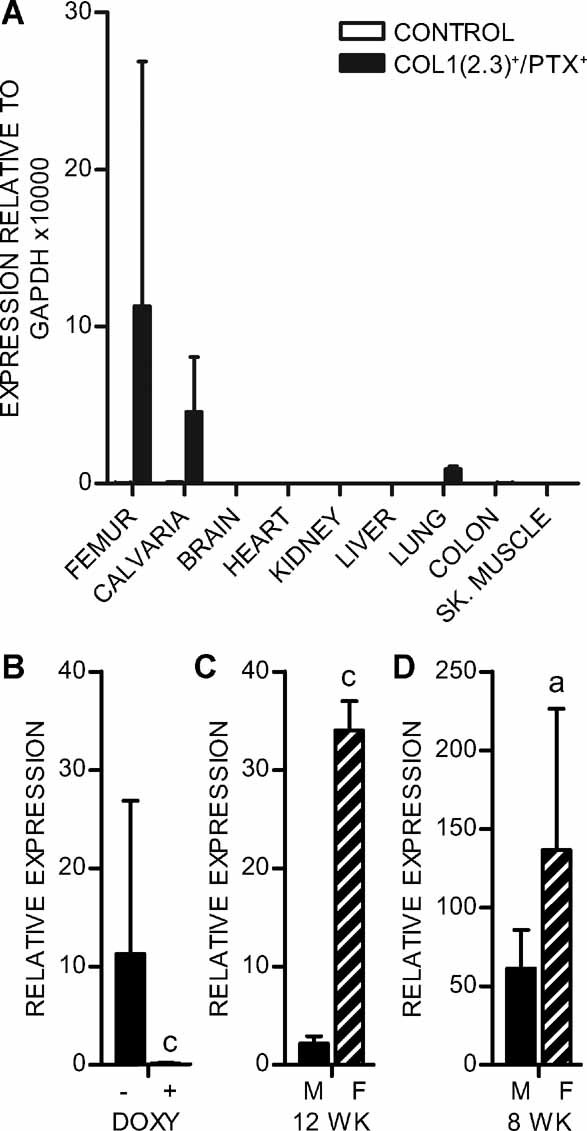
*Col1(2.3)^+^/PTX^+^* mice have bone-specific expression of PTX that is suppressed by administration of doxycycline. PTX expression was assessed in (*A*) a range of tissues isolated from 12-week-old *Col1(2.3)^+^/PTX^+^* mice and littermate controls continuously maintained in the absence of doxycycline, (*B*) femurs derived from 12-week-old *Col1(2.3)^+^/PTX^+^* mice bred and maintained either in the absence (–DOXY, *n* = 6) or presence (+DOXY, *n* = 8) of doxycycline, (*C*) femurs derived from male (*n* = 4) and female (*n* = 2) 12-week-old *Col1(2.3)^+^/PTX^+^* mice maintained in the absence doxycycline, and (*D*) femurs derived from male (*n* = 6) and female (*n* = 6) 8-week-old *Col1(2.3)^+^/PTX^+^* mice maintained in the absence doxycycline. All data were obtained by RT-qPCR analysis and are expressed as mean ± 1 SD. ^a^*p* < .05; ^c^*p* < .001.

In order to allow for the temporal control of PTX expression, we used the tetracycline transactivator (*tTA*) system (TetOff).(27,28) To obtain spatial control, *TetO-PTX* transgenic mice were mated with transgenic mice expressing *tTA* under the control of the osteoblast-specific *Col1*α*1 2.3-kb promoter fragment.*(7) PTX expression was assessed in a range of tissues from 12-week-old double-transgenic progeny [designated *Col1(2.3)*^*+*^*/PTX*^*+*^] and *Col1(2.3)-tTA* single-transgenic controls (Fig. 1*A*). In animals maintained off doxycycline, expression of PTX is limited to long bones and calvaria, with some off-target expression in the lung. PTX expression was almost completely suppressed in mice maintained on a diet that contained doxycycline (Fig. 1*B*). We observed large variation in PTX expression levels and examined the expression data by gender segregation (Fig. 1*C*). Expression of PTX in bone from 12-week-old males was readily detectable but much lower than that observed for females. When PTX expression levels were assessed in younger animals (8 weeks), they were found to be much higher than in the 12-week-old animals, and the gender difference in expression levels was much less pronounced (Fig. 1*D*).

### Effects of PTX expression on growth

**Fig. 2:**
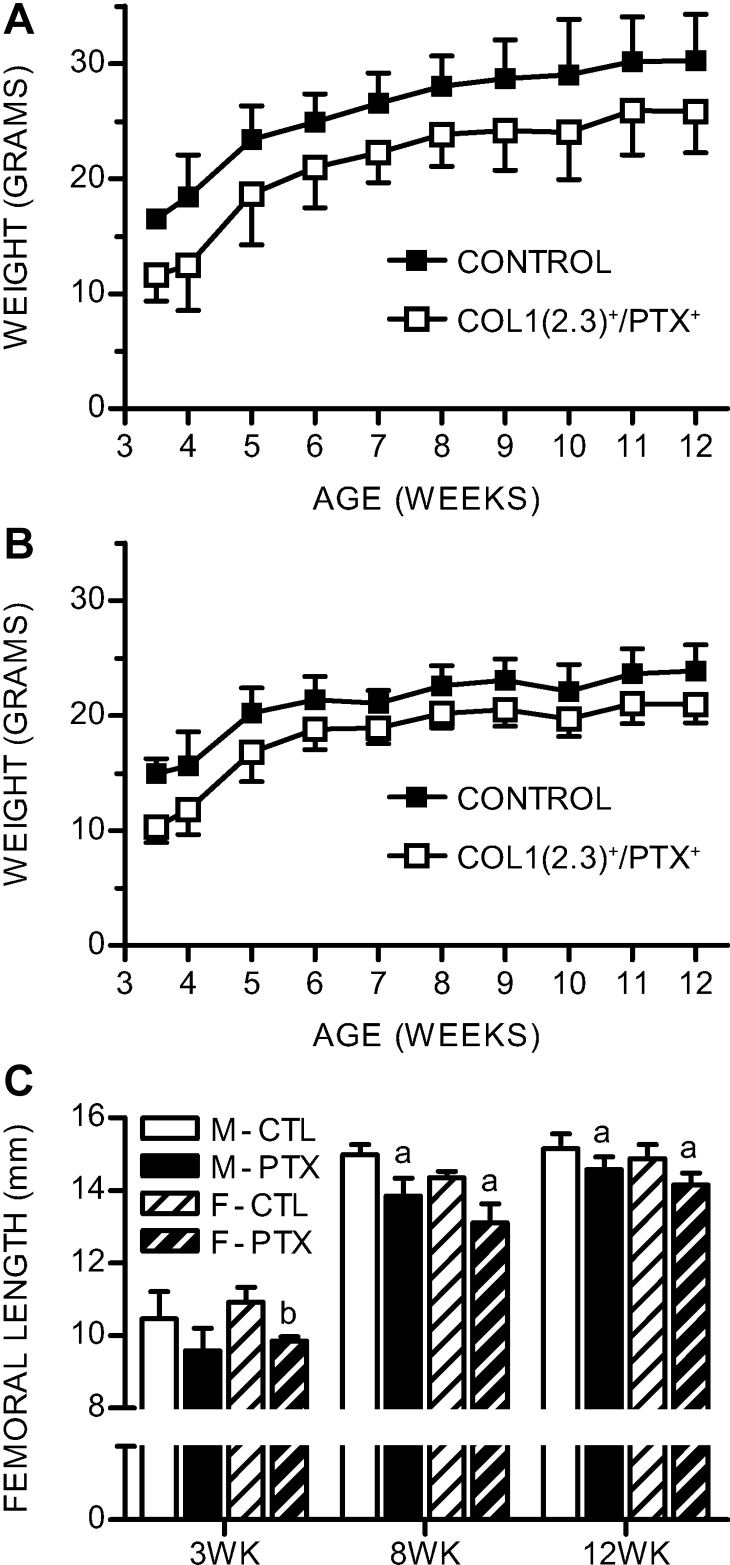
Male and female *Col1(2.3)^+^/PTX^+^* mice are consistently smaller than sex-matched littermate controls and have shorter femoral lengths. Postweaning body weights of (*A*) male and (*B*) female *Col1(2.3)^+^/PTX^+^* mice and littermate controls (*n* ≥ 6 per group). (*C*) Femoral lengths measured following killing at 3 weeks (*n* ≥ 5), 8 weeks (*n* ≥ 10), and 12 weeks of age (*n* ≥ 7). All data are mean ± 1 SD. Statistical significance ascertained compared with age- and sex-matched controls. ^a^*p* < .05; ^b^*p* < .01; ^c^*p* < .001.

*Col1(2.3)*^*+*^*/PTX*^*+*^ mice were consistently runted, with body weights 70% of their sex-matched wild-type littermates at weaning. After weaning, *Col1(2.3)*^*+*^*/PTX*^*+*^ mice displayed healthy weight gain, with male *Col1(2.3)*^*+*^*/PTX*^*+*^ mice weights stabilizing at around 85% of littermate controls and females at 89% of littermate controls. Two-way ANOVA analysis of the growth curves of male (Fig. 2*A*) and female (Fig. 2*B*) mice demonstrated that the effect of the *Col1(2.3)*^*+*^*/PTX*^*+*^ genotype on weight was significant (*p* < .001). This growth phenotype can be blunted by maintaining *Col1(2.3)*^*+*^*/PTX*^*+*^ mice on a doxycycline diet [12-week “on doxycycline” weights, controls versus *Col1(2.3)*^*+*^*/PTX*^*+*^: male 27 ± 4 g versus 25 ± 2 g, *n* = 8, *p* = .37; female 23 ± 3 g versus 22 ± 3 g, *n* = 11, *p* = .31], suggesting that the phenotype depends on the expression of PTX. In addition to reduced body weight, *Col1(2.3)*^*+*^*/PTX*^*+*^ mice maintained in the absence of doxycycline also demonstrate reduced long bone length (Fig. 2*C*).

### Effects of PTX expression on cortical bone

**Fig. 3:**
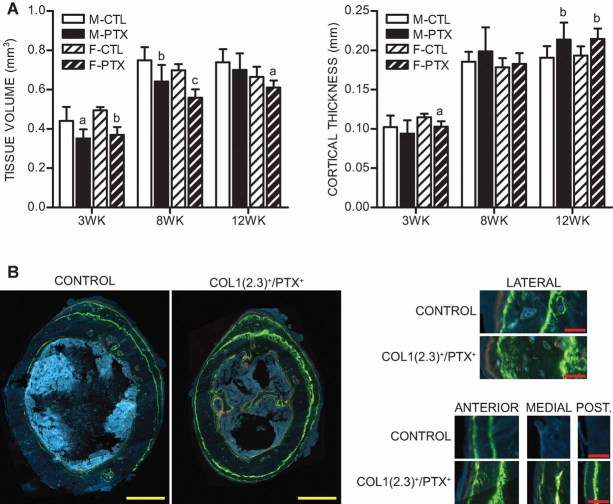
Adult *Col1(2.3)^+^/PTX^+^* mice have increased cortical thickness associated with increased periosteal bone formation. (*A*) Cortical tissue volume and cortical thickness were assessed by µCT at the midshaft of femurs isolated from male (*solid bars*) and female (*striped bars*) mice at 3 weeks (*n* ≥ 5), 8 weeks (*n* ≥ 10), and 12 weeks of age (*n* ≥ 7). All data are mean ± 1 SD. Statistical significance ascertained compared with age- and sex-matched controls. ^a^*p* < .05; ^b^*p* < .01; ^c^*p* < .001. (*B*) Representative images of mid-diaphyseal sections of 8-week-old femurs labeled with demeclocycline 2 days prior to killing and with calcein at 1 and 3 weeks prior to killing. Higher-power images are provided of the lateral, anterior, medial, and posterior periosteal surfaces. Scale bars in yellow are 500 µm. Scale bars in red are 50 µm.

Cortical bone of *Col1(2.3)*^*+*^*/PTX*^*+*^ mice maintained in the absence of doxycycline was assessed by µCT at the femoral midshaft (Fig. 3). In young animals (3 weeks old), both the tissue volume and the cortical thickness were decreased in *Col1(2.3)*^*+*^*/PTX*^*+*^ mice, consistent with their smaller stature. However, in older animals, the proportional difference in tissue volume between control and *Col1(2.3)*^*+*^*/PTX*^*+*^ mice was lessened, and at 12 weeks of age, cortical thickness actually was greater in *Col1(2.3)*^*+*^*/PTX*^*+*^ mice than in controls (Fig. 3*B*). This phenomenon, which was observed in both male and female mice, was further examined by dynamic histomorphometry in 8-week-old animals. The dynamic labeling protocol for the 8-week-old mice was devised prior to full characterization of the femoral midshaft phenotype in younger animals and was designed to cover as broad a developmental period as possible. A dramatic increase in the periosteal bone-formation rate (BFR/BS) resulting from increased mineralizing surface was observed in *Col1(2.3)*^*+*^*/PTX*^*+*^ mice when assessed using a 5-day labeling interval between a calcein label given at 7 weeks of age and a demeclocycline label given at 2 days before euthanasia (Table 1). Similar results were observed using a longer labeling interval (calcein label given at 5 and 7 weeks of age; data not shown). Fluorescence microscopy of femoral midshaft sections demonstrate that the pattern of periosteal bone formation is significantly altered in *Col1(2.3)*^*+*^*/PTX*^*+*^ mice (Fig. 3*B*). Mineralizing surface in control mice was restricted primarily to the lateral and anterior surfaces of the femoral midshaft, with little or no active bone formation on the medial and posterior surfaces. In contrast, *Col1(2.3)*^*+*^*/PTX*^*+*^ mice displayed an expanded mineralizing surface with active bone formation evident around the entire circumference of the bone. While there are clear regional differences in mineral apposition rate (MAR) on the periosteal surfaces of both control and *Col1(2.3)*^*+*^*/PTX*^*+*^ bones, the aggregate MAR across the entire periosteal surface was not altered in *Col1(2.3)*^*+*^*/PTX*^*+*^ mice. Quantitation of endocortical bone formation gave less striking results. While there was a suggestion of increased endocortical mineralizing surface (significant only in females) and decreased endocortical MAR (significant only in males), no change in endocortical BFR/BS was observed (Table 2).

**Table 1:** Periosteal Dynamic Histomorphometric Measurements Performed on *Col1(2.3)*^+^*/PTX*^+^ Mice and Littermate Controls at the Femoral Midshaft

Mice	MS/BS (%)	MAR (µm/day)	BFR/BS (µm^3^/µm^2^/day)
Male, control	50 ± 7	2.6 ± 0.4	1.3 ± 0.3
Male, *Col1(2.3)*^*+*^*/PTX*^*+*^	75 ± 13^c^	2.7 ± 0.6	2.0 ± 0.6^b^
Female, control	45 ± 5	2.6 ± 0.5	1.2 ± 0.3
Female, *Col1(2.3)*^*+*^*/PTX*^*+*^	73 ± 15^c^	2.7 ± 0.7	2.0 ± 0.8^a^

*Note:* Measurements of percent mineralizing surface (MS/BS), mineral apposition rate (MAR), and bone-formation rate (BFR/BS) were performed on femurs of 8-week-old mice, male (*n* = 10) and female (*n* = 8). Statistically significant changes relative to the corresponding littermate control group are indicated by ^a^*p* < .05, ^b^*p* < .01, or ^c^*p* < .001.

**Table 2:** Endocortical Dynamic Histomorphometric Measurements Performed on *Col1(2.3)*^*+*^*/PTX*^*+*^ Mice and Littermate Controls at the Femoral Midshaft

Mice	MS/BS (%)	MAR (µm/day)	BFR/BS (µm^3^/µm^2^/day)
Male, control	63 ± 7	1.2 ± 0.3	0.7 ± 0.2
Male, *Col1(2.3)*^*+*^*/PTX*^*+*^	68 ± 10	0.9 ± 0.2^a^	0.6 ± 0.1
Female, control	77 ± 11	1.5 ± 0.3	1.2 ± 0.4
Female, *Col1(2.3)*^*+*^*/PTX*^*+*^	88 ± 6^a^	1.4 ± 0.3	1.2 ± 0.3

*Note:* Measurements of percent mineralizing surface (MS/BS), mineral apposition rate (MAR), and bone-formation rate (BFR/BS) were performed on femurs of 8-week-old mice, male (*n* = 10) and female (*n* = 8). Statistically significant changes relative to the corresponding littermate control group are indicated by ^a^*p* < .05.

### Effects of PTX expression on cancellous bone structure

**Fig. 4:**
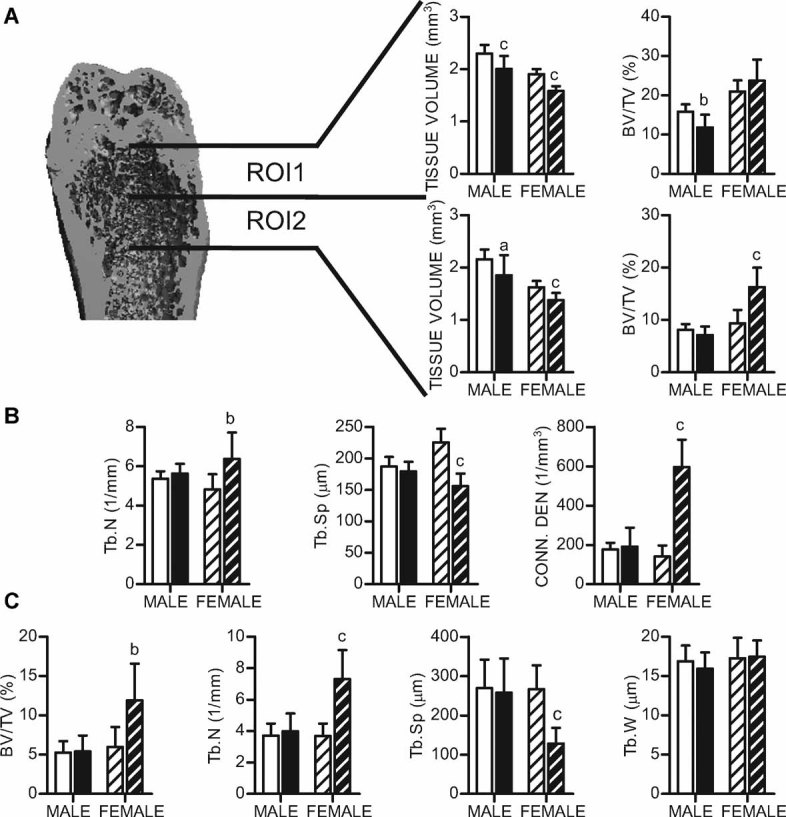
*Col1(2.3)^+^/PTX^+^* mice display a sexually dimorphic cancellous bone phenotype. (*A*) Cancellous bone was assessed by µCT at two volumetric regions of interest, ROI1 and ROI2, of 12-week-old femurs. Tissue volume and fractional bone volume data are provided for both regions. (*B*) Structural parameters derived from the analysis of µCT ROI2. (*C*) Histomorphometric analysis of 12-week-old femurs. All data are mean ± 1 SD; *n* ≥ 9 per group. Statistical significance ascertained compared with age- and sex-matched controls. ^a^*p* < .05; ^b^*p* < .01; ^c^*p* < .001. BV/TV = bone volume/tissue volume; Tb.N = trabecular number; Tb.Sp = trabecular spacing; Conn.Den = connectivity density; Tb.W = trabecular width.

To assess cancellous bone mass in *Col1(2.3)*^*+*^*/PTX*^*+*^ mice, femurs from 12-week-old mice were examined using both µCT and histomorphometry. Assessment using µCT was used initially to evaluate a metaphyseal trabecular ROI immediately adjacent to the primary spongiosa: ROI1 (Fig. 4*A*). The cancellous tissue volume within this region showed a small, statistically significant decrease in *Col1(2.3)*^*+*^*/PTX*^*+*^ mice of both genders, consistent with the smaller size of these animals. Male *Col1(2.3)*^*+*^*/PTX*^*+*^ mice displayed a reduction in fractional bone volume (BV/TV) in ROI1, whereas female *Col1(2.3)*^*+*^*/PTX*^*+*^ mice show no significant change (Fig. 4*A*). Histomorphometric analysis revealed a different story, with male *Col1(2.3)*^*+*^*/PTX*^*+*^ mice displaying no change in BV/TV, whereas female *Col1(2.3)*^*+*^*/PTX*^*+*^ mice displayed a striking increase in BV/TV associated with increased trabecular number, decreased trabecular spacing, and no change in trabecular width (Fig. 4*C*). When considering the contradictory nature of the µCT evaluation of ROI1 and histomorphometric analysis of the same bones, it was noted that the histomorphometric region of analysis was placed further from the growth plate than µCT ROI1. To account for this, the µCT scans were retrieved, and an ROI further from the growth plate, ROI2, was evaluated. The assessment of ROI2 was found to concur with the histomorphometric assessment. Male *Col1(2.3)*^*+*^*/PTX*^*+*^ mice showed no significant changes in BV/TV, and female *Col1(2.3)*^*+*^*/PTX*^*+*^ mice had strikingly increased BV/TV (Fig. 4*A*). The female-specific increase in BV/TV in ROI2 was associated with an increase in trabecular number, decrease in trabecular spacing, and increased connectivity density (Fig. 4*B*). In summary, the phenotype of *Col1(2.3)*^*+*^*/PTX*^*+*^ mice with respect to cancellous bone volume was found to be both sexually dimorphic and dependent on the metaphyseal trabecular ROI chosen for analysis.

### Effects of PTX expression on osteoblasts and bone formation

**Fig. 5:**
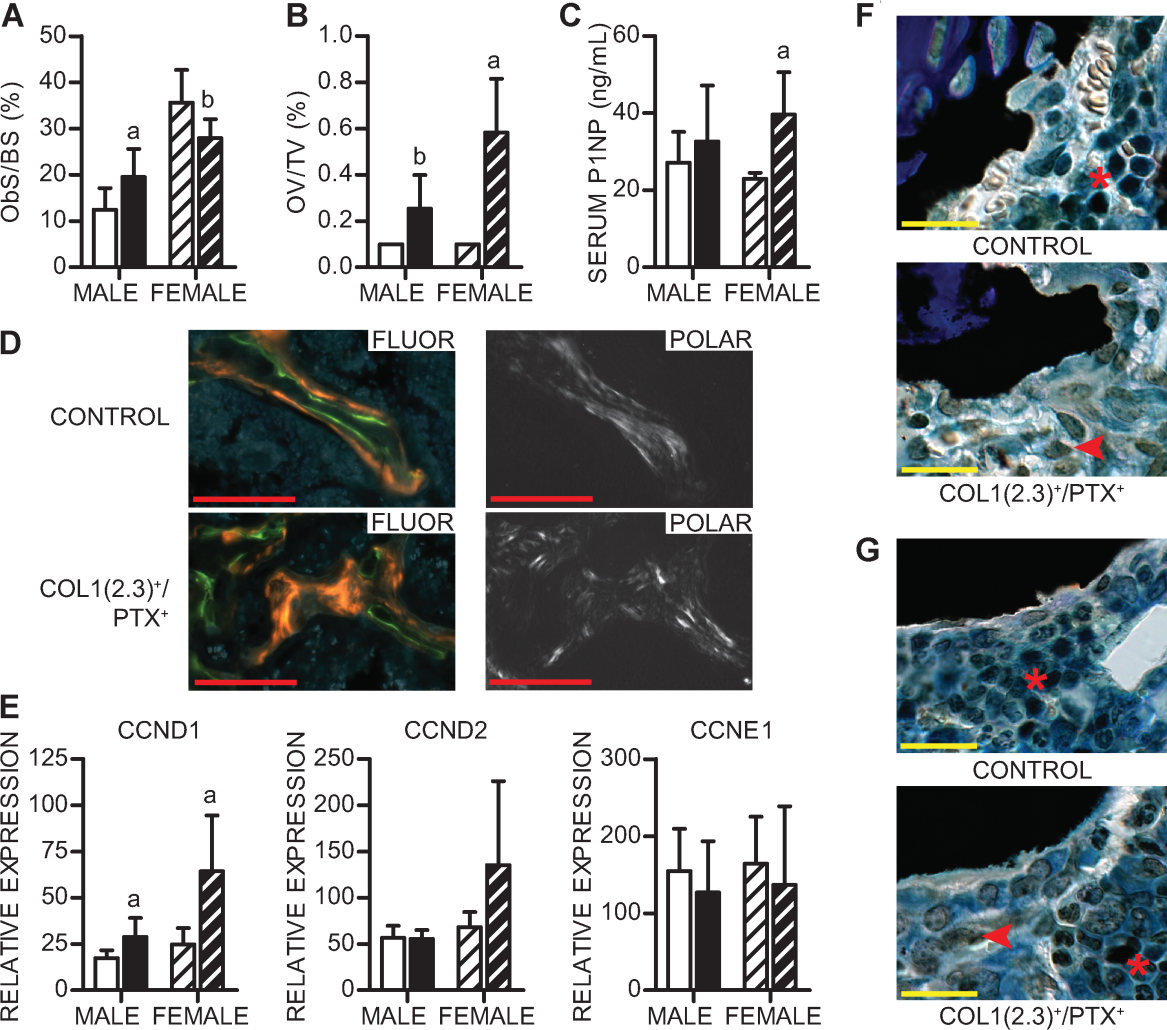
Female *Col1(2.3)^+^/PTX^+^* mice show evidence of increased osteoblast activity. (*A*) Osteoblast surface per bone surface (Ob.S/BS) assessed by histomorphometry in sections of 12-week-old femurs (*n* ≥ 9 per group). (*B*) Fractional osteoid volume (OV/TV; osteoid volume/tissue volume) assessed in 12-week-old femurs (*n* ≥ 7 per group). (*C*) Serum N-terminal propeptide of type I procollagen (PINP), a marker of osteoblast activity, measured in serum from 8-week-old animals (*n* = 4 per group). (*D*) Representative images of cancellous bone from the distal femurs of 8-week-old female mice observed under fluorescent (Fluor) or polarized (Polar) light. Mice received injections of label at 1 week (calcein) and 2 days (demeclocycline) prior to killing. Scale bars in red are 100 µm. (*E*) Expression level in bone of cyclins: CCND1 = cyclin D1; CCND2 = cyclin D2, CCNE1 = cyclin E1. All expression data were obtained by RT-qPCR analysis of RNA isolated from 8-week-old femurs (*n* = 6 per group). All data are shown as mean ± 1 SD. Statistical significance ascertained compared with age- and sex-matched controls. ^a^*p* < .05; ^b^*p* < .01. (*F*) Representative images of von Kossa– and tetrachrome-stained primary spongiosa from the distal femurs of 12-week-old mice. (*G*) Representative images of von Kossa– and tetrachrome-stained secondary spongiosa from the distal femurs of 12-week-old mice. Marrow elements are marked with an asterisk. Osteoblast-like cells not associated with the bone surface are marked with an arrowhead. Scale bars in yellow are 20 µm.

Consistent with the sexually dimorphic changes in cancellous bone mass, levels of serum N-terminal propeptide of type I procollagen (PINP), a marker of bone formation, were increased in female *Col1(2.3)*^*+*^*/PTX*^*+*^ mice compared with littermate controls, but not in male *Col1(2.3)*^*+*^*/PTX*^*+*^ mice (Fig. 5*C*). However, sexually dimorphic changes observed in osteoblast surface did not correlate with changes in cancellous bone mass, with female *Col1(2.3)*^*+*^*/PTX*^*+*^ mice showing decreased osteoblast surface and males showing increased osteoblast surface (Fig. 5*A*). These changes in osteoblast surface also did not correlate with the lack of alteration in the expression of osteoblast marker genes *Runx2*, *osterix*, and *osteocalcin* in RNA isolated from 8-week-old femurs (data not shown). Curiously, both male and female *Col1(2.3)*^*+*^*/PTX*^*+*^ mice demonstrated an increase in osteoid volume. Quantifiable amounts of osteoid are not typically observed in the mouse, and all control animals were assigned a minimum detectable osteoid volume of 0.1%, whereas osteoid was readily quantifiable in all samples of *Col1(2.3)*^*+*^*/PTX*^*+*^ mice (Fig. 5*B*). Alterations in cellular morphology also were noted in both male and female *Col1(2.3)*^*+*^*/PTX*^*+*^ mice. *Col1(2.3)*^*+*^*/PTX*^*+*^ mice displayed a notable increase in plump osteoblast-like cells that were not directly associated with the bone surface both in the primary spongiosa (Fig. 5*F*) and in the secondary spongiosa (Fig. 5*F*). This was particularly evident in the primary spongiosa, where in *Col1(2.3)*^*+*^*/PTX*^*+*^ mice marrow elements were never observed within 100 µm of the growth plate. Marrow elements were readily observed in this region in control mice.

To assess whether the increased cancellous bone mass observed in female *Col1(2.3)*^*+*^*/PTX*^*+*^ mice could be due to increased osteoblast activity, dynamic histomorphometry was performed in 8-week-old femurs. However, BFR/BS in female *Col1(2.3)*^*+*^*/PTX*^*+*^ bones could not be quantified owing to the disorganized nature of bone formation, which displayed a diffuse labeling pattern consistent with the rapid formation of woven bone (Fig. 5*D*). Polarized light microscopy confirmed the presence of short, disorganized collagen fibers in female *Col1(2.3)*^*+*^*/PTX*^*+*^ cancellous bone compared with cancellous bone from control littermates (Fig. 5*D*). In contrast, expression of PTX produced no alteration in BFR/BS in male mice (control 0.68 ± 0.13 µm^3^/µm^2^/d versus *Col1(2.3)*^*+*^*/PTX*^*+*^ 0.77 ± 0.12 µm^3^/µm^2^/d, *n* = 5, *p* = .30). Curiously, the increased osteoblast activity demonstrated in female *Col1(2.3)*^*+*^*/PTX*^*+*^ mice in vivo could not be replicated in in vitro cultures of primary bone marrow stromal cells (BMSCs) from these mice, as assessed by the number of alkaline phosphatase– and Von Kossa–stained colonies and osteoblast marker gene expression (data not shown). Neither was there a change in the in vitro behavior of male *Col1(2.3)*^*+*^*/PTX*^*+*^ BMSCs (data not shown).

It has been shown previously that treatment of mice with the serotonin synthesis inhibitor pCPA leads to increased expression of cyclin D1, cyclin D2, and cyclin E1 in bone, presumably through reducing activation of the G_i_-coupled Htr1b receptor in osteoblasts.(23) Consequently, we examined the expression of these genes in RNA isolated from 8-week-old femurs in our model of osteoblast-specific G_i_ signaling blockade. Cyclin D1 was significantly increased in both male and female *Col1(2.3)*^*+*^*/PTX*^*+*^ mice, cyclin D2 showed a trend toward being increased in females, and no change was seen in cyclin E1 (Fig. 5*E*).

### Effects of osteoblast-specific PTX expression on osteoclasts

**Fig. 6:**
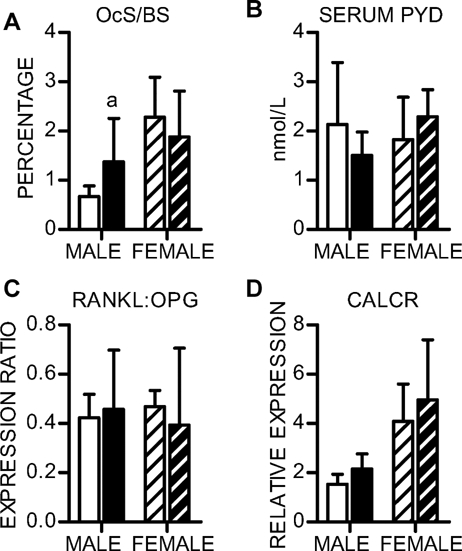
*Col1(2.3)^+^/PTX^+^* mice show little evidence of altered osteoclast activity. (*A*) Osteoclast surface per bone surface (Oc.S/BS) assessed by histomorphometry in sections of 12-week-old femurs (*n* ≥ 9 per group). (*B*) Serum pyridinoline cross-links, a marker of osteoclast activity, measured in sera from 8-week-old animals (*n* = 4 per group). (*C*) The RNA expression ratio of the osteoclastogenic factor *RANKL* and its decoy receptor *OPG* in bone. (*D*) Expression level of the osteoclast marker gene *calcitonin receptor* (*CALCR*) in bone. All expression data were obtained by RT-qPCR analysis of RNA isolated from 8-week-old femurs (*n* = 6 per group). All data are shown as mean ± 1 SD. Statistical significance ascertained compared with age- and sex-matched controls. ^a^*p* < .05.

Whereas osteoblasts have a well-appreciated role in regulating osteoclast number and activity, we saw little evidence suggesting that blockade of G_i_-coupled signaling in osteoblasts has downstream effects on osteoclasts. Histomorphometry revealed an increase in osteoclast surface only in *Col1(2.3)*^*+*^*/PTX*^*+*^ males (Fig. 6*A*). However, levels of serum pyridinoline cross-links, a marker of bone resorption, were unaltered in both male and female *Col1(2.3)*^*+*^*/PTX*^*+*^ mice (Fig. 6*B*). RANKL and osteoprotegerin (OPG) are important factors expressed by osteoblasts that regulate osteoclastogenesis.(29) Expression of RANKL and OPG and the ratio of RANKL/OPG expression remain unchanged by osteoblast-specific PTX expression in bone from 8-week-old femurs (Fig. 6*C* and data not shown). Similarly, expression of the calcitonin receptor, a gene highly expressed in osteoclasts, is unchanged in bone from *Col1(2.3)*^*+*^*/PTX*^*+*^ mice (Fig. 6*D*).

## Discussion

For several decades, sensitivity to pertussis toxin has been the “gold standard” in determining the involvement of receptor-activated G_i_ signaling in in vitro assays. Here we describe the osteoblast-specific, tetracycline-regulated expression of the S_1_ catalytic subunit of pertussis toxin for the purpose of probing the role of this signaling pathway in vivo. Previously, transgenic expression of PTX has been used in vivo to assess the role of G_i_ signaling in T cells,(30) and a model for *Cre*-inducible PTX expression has been demonstrated in pancreatic β cells.(31) Our model is unique in that it allows for the reversible regulation of PTX expression. For this to be significant, it is imperative that suppression of PTX expression by doxycycline is sufficient to ablate the functional enzymatic activity of PTX. Our observation that maintaining *Col1(2.3)*^*+*^*/PTX*^*+*^ mice on a doxycycline diet suppresses the growth phenotype of these animals suggests that this is the case. As in other models of in vivo PTX expression, we saw no evidence that chronic expression of PTX negatively affected cell health. *Col1(2.3)*^*+*^*/PTX*^*+*^ mice showed normal skeletal development and, in fact, demonstrated site-specific increases in osteoblast activity.

Examination of the role of individual G_i_-coupled GPCRs in regulation of skeletal homeostasis has yielded evidence suggesting that G_i_ signaling in osteoblasts may have either anabolic or catabolic effects in bone. The phenotype of the *Col1(2.3)*^*+*^*/PTX*^*+*^ mouse clearly demonstrates that the predominant role of G_i_-coupled signaling by endogenous receptors in mature osteoblasts is to restrict bone formation. However, blockade of G_i_-coupled signaling by endogenous receptors does not universally affect the bone-forming activity of all osteoblasts. Rather, regional specificity in where bone formation was increased was observed in *Col1(2.3)*^*+*^*/PTX*^*+*^ mice. This suggests that osteoblasts at different sites may be distinguished from each other either by the complement of G_i_-coupled receptors that they express or by differential exposure to the ligands of these receptors. In fact, this may be one mechanism by which cortical drift, the differential regulation of bone formation at different cortical surfaces, is normally established. The phenomenon of cortical drift is poorly understood but is of clear importance in determining bone shape as well as bone volume. The role of cortical drift in regulating bone shape is recognized both during growth(32–34) and in response to mechanical stimuli.(35,36) *Col1(2.3)*^*+*^*/PTX*^*+*^ mice do not have gross deformities in bone development, patterning, or shape, but the altered pattern of bone formation does suggest that they will develop changes in femoral shape as they age. The effect of these changes on the biomechanical properties of the bone remains to be investigated.

Regional specificity of increases in bone formation also was observed in cancellous bone of *Col1(2.3)*^*+*^*/PTX*^*+*^ mice. Assessment of the secondary spongiosa at the distal femur conducted in two ROIs using µCT yielded significantly different results in the two regions. In ROI1, close to the primary spongiosa, fractional bone volume was decreased or unchanged in *Col1(2.3)*^*+*^*/PTX*^*+*^ mice, whereas in ROI2, further from the growth plate and primary spongiosa, fractional bone volume was unchanged or increased in *Col1(2.3)*^*+*^*/PTX*^*+*^ mice. Our data suggest that there may be a differential role in G_i_ signaling in newer bone formed in ROI1 compared with more mature bone found in ROI2. Our data also describe a sexually dimorphic cancellous bone phenotype in *Col1(2.3)*^*+*^*/PTX*^*+*^ mice. Interpretation of this sexual dimorphism is complicated by our observation of differential levels of PTX expression in bone from male and female mice. PTX is an enzyme and would be expected to yield complete blockade of receptor-mediated G_i_ activation in osteoblasts at low levels of expression. However, owing to difficulties in obtaining a homogeneous population of *Col1(2.3)*-defined osteoblasts for biochemical analysis, we have been unable to directly assess the efficacy of the G_i_ signaling blockade. Hence we are currently unable to conclude that osteoblast-specific G_i_ signaling blockade was achieved equally in males and females. It is also unclear whether the difference in PTX expression in males and females represents a genuine gender difference in the activity of the mouse *Col1(2.3)* promoter. Alternatively, a sexually dimorphic response to the expression of PTX could lead to maintenance of higher levels of *Col1(2.3)* promoter activity in females but not males as the animals age. In favor of the sexually dimorphic phenotype resulting from a differential response to blockade of receptor-activated G_i_ signaling, we offer the following observations: (1) At 8 weeks of age, the difference in expression of PTX in bone between males and females is small, yet there is a great difference between males and females in the uptake of calcein labels for the assessment of bone formation. All male *Col1(2.3)*^*+*^*/PTX*^*+*^ mice had quantifiable labels, whereas all female *Col1(2.3)*^*+*^*/PTX*^*+*^ mice demonstrated smeared, disorganized label that could not be quantified. (2) Although male *Col1(2.3)*^*+*^*/PTX*^*+*^ mice did not differ from littermate controls in their cancellous BFR/BS or histomorphometrically assessed fractional bone volume, they are not completely devoid of a cancellous bone phenotype. Histology and histomorphometry of the distal femur showed male *Col1(2.3)*^*+*^*/PTX*^*+*^ mice to have increased osteoid volume and increased number of osteoblast-like cells not directly associated with the bone surface. Increased osteoid volume in males in the absence of altered BFR/BS or BV/TV suggests that there may be a role for G_i_ signaling promoting normal bone mineralization. Increased osteoid volume seen in female *Col1(2.3)*^*+*^*/PTX*^*+*^ mice may be contributed to by the rapid rate of bone formation leading to a lag in mineralization of bone matrix.

Despite the in vivo phenotype of *Col1(2.3)*^*+*^*/PTX*^*+*^ mice, we could not demonstrate altered behavior of primary BMSC cultures derived from these mice. The absence of correlation between the in vivo and in vitro behavior of *Col1(2.3)*^*+*^*/PTX*^*+*^ osteoblasts could result from a number of possible causes. First, we postulate that the *Col1(2.3)*^*+*^*/PTX*^*+*^ mouse phenotype results from the inhibition of a G_i_-coupled signal in osteoblasts. If the ligand that stimulates this signal is missing in vitro, we would expect a loss of phenotype in vitro. We have reported previously that *Col1(2.3)*^*+*^*/Ro1*^*+*^ mice, which have osteoblast-specific expression of a constitutively active G_i_-coupled engineered receptor, display osteopenia in vivo. However, BMSC and calvarial cultures of cells derived from *Col1(2.3)*^*+*^*/Ro1*^*+*^ mice also do not show any overt phenotype in vitro(7) (data not shown). Hence neither inhibition nor activation of G_i_-coupled signaling in osteoblasts in vitro results in a phenotype, despite significant in vivo phenotypes. This suggests that some other component of the bone microenvironment necessary for manifestation of the phenotypes resulting from altered G_i_-coupled signaling in osteoblasts in vivo is missing from the in vitro culture systems.

We had hypothesized that the role of G_i_ signaling in osteoblasts may be to antagonize increases in cAMP levels in response to G_s_-coupled signaling. If this were the case, blockade of receptor-mediated G_i_ signaling in osteoblasts would be expected to yield a similar phenotype to models of increased G_s_ signaling in osteoblasts. The phenotypes of transgenic mouse models with osteoblast-specific activation of G_s_-coupled signaling typically are characterized by increased trabecular bone formation, marrow fibrosis, and cortical bone resorption.(4–6) This differs significantly from the *Col1(2.3)*^*+*^*/PTX*^*+*^ model, where increased trabecular bone formation is noted only in female mice. While there is an expansion of osteoblast-like cells not directly associated with the bone surface in both male and female *Col1(2.3)*^*+*^*/PTX*^*+*^ mice, this model does not display the marked areas of marrow fibrosis observed in models of increased G_s_ signaling in osteoblasts. Additionally, decreased cortical thickness and dramatically reduced periosteal MAR and BFR/BS in models of increased G_s_ signaling in osteoblasts(5) contrast with the increased cortical thickness, periosteal mineralizing surface, and BFR/BS in the *Col1(2.3)*^*+*^*/PTX*^*+*^ mouse. Increased G_s_-coupled signaling in osteoblasts is also classically associated with increased RANKL expression and increased osteoclastogenesis and activity. We saw no increase in RANKL expression in bone and no indication of increased osteoclast activity in *Col1(2.3)*^*+*^*/PTX*^*+*^ mice. These data suggest that while G_s_-coupled signaling in osteoblasts promotes bone formation and G_i_-coupled signaling in osteoblasts restricts bone formation, the downstream consequences of blockade of G_i_-coupled signaling in osteoblasts are not exclusively mediated by relieving inhibition of adenylyl cyclase–mediated production of the second-messenger cAMP.

Our data suggest that receptor-activated G_i_ signaling in osteoblasts acts as a site-specific constitutive negative regulator of bone and may be a therapeutic target. In recent years, another class of constitutive negative regulators of bone formation, the secreted Wnt inhibitors, has been fruitfully targeted in the search for anabolic therapies for osteoporosis. While there are many secreted Wnt inhibitors, they appear not to be functionally redundant, and antagonism of a single inhibitor yields increases in bone formation. Perhaps the same will be true for G_i_-coupled GPCRs, and identifying the full complement of G_i_-coupled GPCRs expressed in osteoblasts may reveal novel therapeutic targets. The G_i_-coupled serotonin receptor Htr1b is one such target, and suppression of gut-derived serotonin synthesis has shown promise in treating rodent models of postmenopausal bone loss.(37) Provided that the increased bone formation and bone volume associated with the blockade of G_i_-coupled signaling can be shown to correlate with increased bone strength, it represents a novel therapeutic target for the development of bone anabolic therapies.
