# Supplementary material for: Blockade of Receptor-Activated Gi Signaling in Osteoblasts In Vivo Leads to Site-Specific Increases in Cortical and Cancellous Bone Formation
Source: J Bone Miner Res. 2010 Oct 11;26(4):822–32. doi: 10.1002/jbmr.273 (PMC3179326; doi:10.1002/jbmr.273)
Supplement: Supplementary file 1 [file jbmr0026-0822-SD1.docx]

**Supplemental Data**

**Table S1:** Sequences of primers used for real-time PCR with SYBR green chemistry

| Gene | 5’ primer | 3’ primer | Genbank |
| --- | --- | --- | --- |
| Calcitonin Receptor | tccagagtgaaaaggcggaat | aacctgttcacgagaaggaacct | NM_001042725 |
| Collagen Type1 | gcgaaggcaacagtcgct | cttggtggttttgtattcgatgac | U08020 |
| Dkk1 | ccgggaactactgcaaaaat | cgttgtggtcattaccaagg | AF030433 |
| PTX | cacaccggcgcattcc | ttgtgatagacccgcgttacc | AJ007364.1 |
| OPG | cagagaccaggaaatggtgaa | aagctgctctgtggtgaggt | NM_008764 |
| Osteocalcin | ctgacctcacagatgccaag | gtagcgccggagtctgttc | NM_007541 |
| Osterix | tttctcattaactcgttgccatct | cttcgggaaaacggcaaata | NM_130458.3 |
| RANKL | ttgcacacctcaccatcaat | tccgttgcttaacgtcatgt | NM_011613 |
| Runx2 | cgagaccaaccgagtcattt | acgccatagtccctcctttt | NM_009821 |
| SOST | accgggcggagaatgg | gctgtactcggacacatctttgg | NM_024449.4 |
| GAPDH | tgcaccaccaactgcttag | ggatgcagggatgatgttc | NM_001001303 |
| L19 | gagtcccggaagacgagag | atcggaagcacctttctcct | NM_026490 |

**Table S2:** Taqman primers used for real-time PCR

| Gene | Applied Biosystems Cat# | Exon Boundary | Genbank |
| --- | --- | --- | --- |
| Axin2 | Mm01265783_m1 | 9-10 | NM_015732.4 |
| CyclinD1 | Mm00432359_m1 | 3-4 | NM_007631.2 |
| CyclinD2 | Mm00438070_m1 | 1-2 | NM_009829.3 |
| CyclinE1 | Mm00432367_m1 | 4-5 | NM_007633.2 |
| Lef1 | Mm00550265_m1 | 3-4 | NM_010703.3 |
| GAPDH | Mm99999915_g1 | 2-3 | NM_008084.2 |
| L19 | Mm02601633_g1 | 5-6 | NM_001159483.1 |
